# Alpha-Glucosidase- and Lipase-Inhibitory Phenalenones from a New Species of *Pseudolophiostoma* Originating from Thailand

**DOI:** 10.3390/molecules25040965

**Published:** 2020-02-20

**Authors:** Allan Patrick G. Macabeo, Luis Agustin E. Pilapil, Katherine Yasmin M. Garcia, Mark Tristan J. Quimque, Chayanard Phukhamsakda, Allaine Jean C. Cruz, Kevin D. Hyde, Marc Stadler

**Affiliations:** 1 Laboratory for Organic Reactivity, Discovery and Synthesis (LORDS), Research Center for the Natural and Applied Sciences, University of Santo Tomas, Espana Blvd., Manila 1015, Philippines; allanpatrick_m@yahoo.com (A.P.G.M.); luisagustinpilapil@gmail.com (L.A.E.P.); kymgarcia_1128@yahoo.com (K.Y.M.G.); mtjquimque@gmail.com (M.T.J.Q.); allainecruzz@gmail.com (A.J.C.C.); 2 Department of Microbial Drugs, Helmholtz Centre for Infection Research and German Centre for Infection Research (DZIF), partner site Hannover/Braunschweig, Inhoffenstrasse 7, 38124 Braunschweig, Germany; 3 Center of Excellence in Fungal Research, Mae Fah Luang University, Chiang Rai 57100, Thailand; chayanard91@gmail.com (C.P.); kdhyde3@gmail.com (K.D.H.)

**Keywords:** Dothideomycetes, *Pseudolophiostoma* sp., phenalenones, anti-α-glucosidase, anti-lipase, molecular docking

## Abstract

The alpha-glucosidase- and lipase-inhibitory activities of three phenalenones (**1**–**3**) and one phenylpropanoid (**4**) from the ethyl acetate extracts of a *Pseudolophiosptoma* sp. are described. They represent the first secondary metabolites reported from the genus *Pseudolophiostoma*. Scleroderolide (**1**) and sclerodione (**2**) exhibited potent α-glucosidase- and porcine-lipase-inhibitory activity during primary screening, with better IC_50_ values compared to the positive controls, N-deoxynojirimycin and orlistat. In silico techniques were employed to validate the probable biological targets and elucidate the mechanism of actions of phenalenones **1** and **2**. Both compounds exhibited strong binding affinities to both alpha-glucosidase and porcine lipase through H-bonding and π–π interactions. Interestingly, favorable in silico ADME (absorption, distribution, metabolism, and excretion) properties such as gastrointestinal absorption were also predicted using software.

## 1. Introduction

Diabetes and obesity are chronic metabolic disorders that are fast increasing worldwide. It has been established that a high body mass index is strongly correlated with diabetes and insulin resistance. Additionally, an increase of metabolic factors involved in the progression of insulin resistance, such as non-esterified fatty acids (NEFA), glycerol, hormones, cytokines, proinflammatory substances, and other related substances, are observed in obese individuals. Thus, gaining weight in early life is associated with the development of type 1 diabetes [1]. Therefore, modern approaches to drug discovery must be applied to prevent diabetes and obesity in individuals [2]. Among the initial targets for discovering new agents against diabetes and obesity are inhibitors of the enzymes α-glucosidase and lipase. Relevant to this study, a number of fungal secondary metabolites have been identified from different taxa with promising inhibitory effects against these two enzymes [3].

The fungal class Dothideomycetes is one of the largest in the Ascomycota and includes many plant pathogens [4]. Dothideomycetes species produce a wide array of secondary metabolites and, in particular, many phytotoxins have been reported to be produced by these species [5]. Recently, biofilm inhibitors of *Staphylococcus aureus* related to abscisic acid from a *Roussoella* sp. and novel spirodioxynaphthalenes with antimicrobial and cytotoxic activities from *Sparticola junci* were reported as part of our ongoing work to explore hitherto untapped tropical genera and species [6,7]. During the course of our biodiversity studies on new Dothideomycetes taxa from Thailand, we came across a new species of *Pseudolophiostoma* (order Pleosporales, family Lophiostomataceae). The genus was only recently introduced [8]. It contains five species [9], which have never before been explored for secondary metabolites and their associated biological activity.

In this paper, we isolated three phenalenone-type polyketides and one phenylpropanoid from *Pseudolophiostoma* sp. (MFLUCC 17-2081), and evaluated their α-glucosidase- and lipase-inhibitory activities. To complement the observed biological activities, molecular docking studies with α-glucosidase and porcine lipase, drug-likeness, and absorption, distribution, metabolism, and excretion (ADME)-Tox studies on the bioactive phenalenones **1** and **2** were also carried out and are reported herein.

## 2. Results and Discussion

*Pseudolophiostoma* sp. was isolated from a dried branch of *Clematis fulvicoma* Rehder & E.H. Wilson collected in Chiang Rai, Thailand in 2017. The fungus formed olive gray colonies on malt extract agar (MEA). The morphological characteristics of strain MFLUCC 17-2081 matched with the generic concept of *Pseudolophiostoma*. However, the results of a multigenic phylogenetic analysis suggested that the fungus represented a distinct species. A formal taxonomic description of the new *Pseudolophiostoma* sp. will be reported elsewhere.

**Figure 1:**
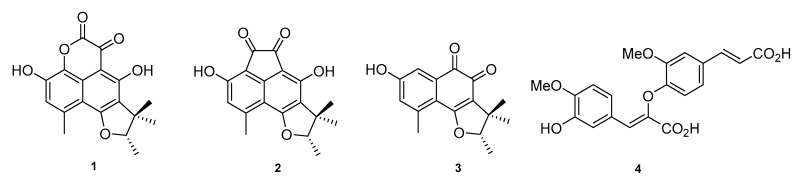
Secondary metabolites isolated from *Pseudolophiostoma clematidis*: scleroderolide (**1**) sclerodione (**2**), trypethelone (**3**) and the phenylpropanoid 8-O-4′-diferulic acid (**4**)

Solid-state fermentation of *Pseudolophiostoma* sp. MFLUCC 17-2081 resulted in luxuriant growth in cooked rice medium. The reddish-orange colored rice cultures were extracted with ethyl acetate to recover the organic metabolites and the resulting crude extract was fractionated using silica gel column chromatography, yielding 10 fractions. Fractions 4 and 6 contained small molecules with interesting HPLC-MS-DAD (high-performance liquid chromatography coupled to mass spectrometry and diode array detection) profiles, and were further purified by reverse-phase (RP)-HPLC to yield four compounds in quantities sufficient for full spectroscopic characterization. Thus, analysis and comparison of spectroscopic data with reported literature data allowed identification of three phenalenone congeners, scleroderolide (**1**) [10,11,12], sclerodione (**2**) [11,13,14,15], and trypethelone (**3**) [14,16], and the phenylpropanoid 8-*O*-4′-diferulic acid (**4**) [17,18] (Figure 1). This is the first report of these secondary metabolites from the genus *Pseudolophiostoma*. Phenalenone derivatives have been previously identified in selected genera of Dothideomycetes, and levorotatory stereoisomers of compounds **1**–**3** have been reported to occur in several fungal species, such as *Coniothyrium cereali* [11], *Gremmeniella abietina* [10], and *Massarina bipolaris* [12].

**Figure 2:**
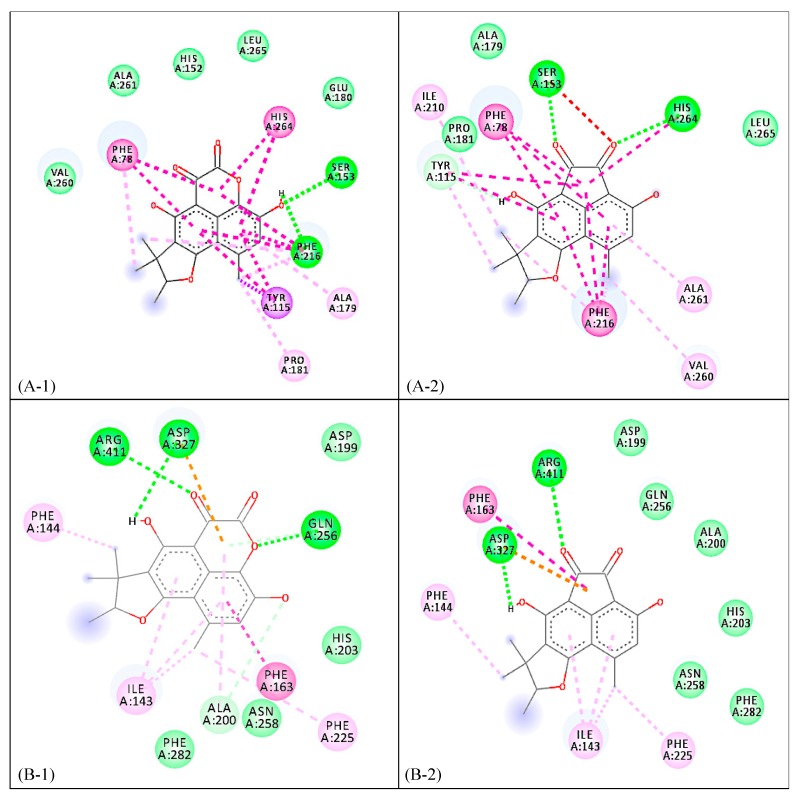
2D binding diagram of **1** and **2** against porcine pancreatic lipase (**A-1** and **A-2**, respectively) and α-glucosidase (**B-1** and **B-2**, respectively).

In terms of the inhibitory properties of phenalenone derivatives 1–3 against the enzymes α-glucosidase and porcine lipase, scleroderolide (**1**) showed the best inhibitory activity (IC_50_ = 48.7 mM) (Table 1) against α-glucosidase among the phenalenone compounds. Interestingly, both **1** and **2** conferred better inhibition than the positive standard, N-deoxynojirimycin (IC_50_ = 130.5 mM). To clarify the inhibitory mechanisms of compounds **1** and **2**, both were docked onto the enzyme α-glucosidase (PDB ID: 5ZCC). The binding energy of **1** with the enzyme was determined to be −9.0 kcal/mol while the binding energy of **2** was calculated as −8.9 kcal/mol. Both **1** and **2** displayed hydrogen bonding between C2-OH, carbonyl oxygen at C5 with Asp^327^ and Arg^411^, respectively. An additional hydrogen bonding can be observed in **1** between its endocyclic lactone oxygen and Gly^256^. Both complexes demonstrated π-π interplay between the conjugated ring system and Phe^163^ residue. Furthermore, the polyaromatic core structures of **1** and **2** analogously showed π-alkyl and π-anion interactions with Ile^143^, Phe^144^, and Phe^225^ which are absent in the reference drug N-deoxynojirimicin (Supplementary Table S1 and Figure 2).

Scleroderolide (**1**) and sclerodione (**2**) were also potently inhibitory of the hydrolysis of p-nitrophenylbutyrate using porcine lipase. Among the phenalenone derivatives, sclerodione (**2**) showed the strongest inhibitory effect (IC_50_ = 1.0 mM), being 9 times more potent than the positive drug control, orlistat (Table 1). To elucidate the mechanism of inhibition of compounds **1** and **2** against the enzyme porcine pancreatic lipase (PDB ID: 1ETH), both compounds were separately complexed in silico to simulate and visualize protein–ligand interactions, which are contributing factors to the affinity and stabilization of a complex. Compound **1** was bound to the enzyme through hydrogen bonding with Ser^153^ and Phe^216^, while **2**, on the other hand, was attached through hydrogen bonding with the catalytic residues of the enzyme [19], Ser^153^ and His^264^ (Supplementary Table S1 and Figure 2). The presence of an aromatic acenaphthylene core strengthened the attachment of **2** to the binding site of the enzyme through π-stacking interactions, particularly with Phe^78^ and Phe^216^ residues. A similar π-stacking interaction was observed between **1** and amino acid residues Phe^78^ and His^264^. However, such π–π interplay was absent in orlistat. All three molecules (**1**, **2**, and orlistat) were involved in π–σ/π–donor hydrogen bonding with Tyr^115^. The approximated binding energies of compounds **1** and **2** against porcine pancreatic lipase were −10.3 kcal/mol and −10.0 kcal/mol, respectively (Supplementary Table S1 and Figure 2).

**Figure 3:**
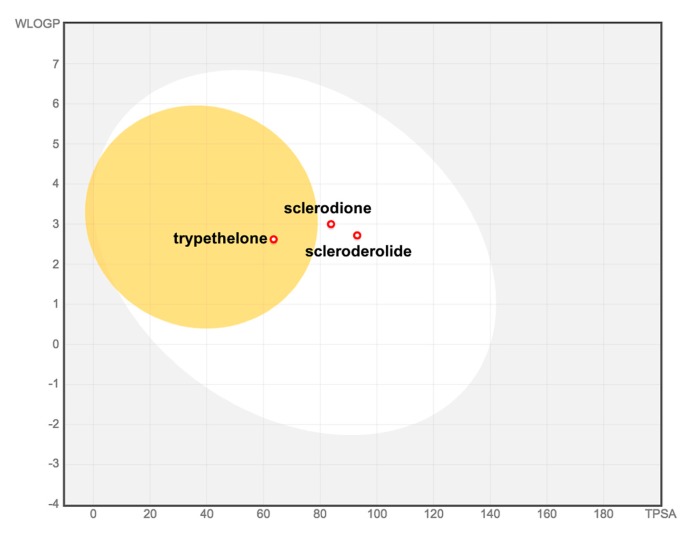
Prediction of gastrointestinal (GI) tract and brain permeation by brain or intestinal estimated permeation predictive model (BOILED-Egg) method.

The two bioactive compounds, **1** and **2**, were also submitted to in silico ADME (absorption, distribution, metabolism, excretion) screening using SwissADME [20] to predict their overall pharmacokinetic behavior (Table 2). The compounds’ druggabilities were assessed based on Lipinski’s (or Pfizer’s) Rule of Five (Ro5) [21], which is focused on the following descriptors: molecular weight (less than 500 Daltons), lipophilicity (octanol–water partition coefficient, logP less than 5), number of hydrogen bond donors (fewer than 10), and acceptors (fewer than 5). Both compounds were found to satisfy Lipinski’s Ro5. Likewise, the BOILED-Egg (brain or intestinal estimated permeation predictive model), which is an intuitive graphical plot of the functions of lipophilicity and apparent polarity (as described by the parameters WLOGP (atomistic octanol-water partition coefficient) and TPSA (topological polar surface area), respectively), was used to predict passive intestinal absorption and brain permeation [22]. Both compounds were predicted to have high gastrointestinal (GI) absorption within acceptable limits (WLOGP > 5.88 and TPSA > 131.6) (Figure 3). However, both had a poor probability of blood–brain barrier (BBB) crossing capacity. Ro5 compliance and a predicted high GI absorption for **1** and **2** theoretically suggest that they may be orally active in humans.

Toxicities and related physicochemical properties, as predicted using the OSIRIS Property explorer program, are presented in Table 3. The predicted toxicity risks observed in **1**–**3** were mostly due to a high-risk 1-substituted naphthalene fragment in their core structures (Table 3). Taking into account all other physicochemical properties, both phenalenones were shown to comply with drug-likeness potentials.

To verify the predicted toxicity of compounds **1**–**3**, a cytotoxicity assessment (Table 4) was performed against mouse fibroblast (L929) and HeLa (KB3.1) cells, as previously reported [23]. Trypethelone (**3**) showed moderately strong cytotoxicity, which correlates well with the predicted reproductive toxicity, for which it was shown to have a high risk of toxicity. Scleroderolide (**1**) and sclerodione (**2**) exhibited weaker activity. Compound **2** was previously shown to possess moderate antimicrobial activities [11], but otherwise no prominent bioactivities of these compounds are known.

## 3. Materials and Methods

### 3.1. General Experimental Procedures

Optical rotation values were determined using a Perkin-Elmer (Überlingen, Germany) 241 spectrophotometer. NMR spectra were recorded on a Bruker (Bremen, Germany) 500 MHz Avance III spectrometer with a BBFO (plus) SmartProbe (^1^H 500 MHz, ^13^C 125 MHz) and Bruker Ascend 700 spectrometer equipped with 5 mm TXI cryoprobe (^1^H-700 MHz, ^13^C-175 MHz) spectrometers, locked to the deuterium signal of the solvent. NMR spectra were measured in acetone-*d_6_*; chemical shifts were referenced to the solvent signals. HR-ESI-MS mass spectra were recorded using a Bruker (Bremen, Germany) Agilent 1260 series HPLC-UV/Vis system (column 2.1 × 50 mm, 1.7 m, C18 Acquity UPLC BEH (waters); solvent A: H_2_O + 0.1% formic acid; solvent B: AcCN + 0.1% formic acid, gradient: 5% B for 0.5 min, increasing to 100% B in 19.5 min and then maintaining 100% B for 5 min, flow rate 0.6 mL/min, UV/Vis detection at 200–600 nm combined with ESI-TOF-MS (Maxis, Bruker, Bremen, Germany) with scan range 100–2500 m/z, capillary voltage 4500 V, dry temperature 200 °C.

### 3.2. Fungal Material

Following standard procedures, the fungus was isolated from *Clematis fulvicoma* Rehder & E.H. Wilson. The plant material was collected in March 2017 in Chiang Rai, Thailand, and the dried herbarium specimen and culture were deposited at Mae Fah Luang culture collections as MFLU 17-2081. The fungus was identified by morphological study and DNA sequence data (5.8S gene region, the internal transcribed spacers ITS1 and ITS2) as a species of *Pseudolophiostoma*. These sequence data were deposited in GenBank with accession number MN393004. A Genomic DNA Miniprep kit (Bio Basic Canada Inc., Markham, ON, Canada) was used. A Precellys 24 homogenizer (Bertin Technologies, Saint-Quentin-en-Yvelines, France) was used for cell disruption, run twice at a speed of 6000 rpm for 40 s. The ITS gene region was amplified with primers ITS5 and ITS4. The fungal strain is kept in the culture collection of Mae Fah Luang University (Chiang Rai, Thailand) with the code number MFLUCC 17-2081. A detailed taxonomic description will be published concurrently.

### 3.3. Fermentation and Extraction

Twenty 1 L Erlenmeyer flasks with solid rice medium (100 g of rice and 110 mL of demineralized water, each) were used for fermentation. They were autoclaved at 121 °C for 2 h and cooled to room temperature, followed by inoculation with the fungus. After cultivation at 20 °C under static conditions for 8 weeks, 500 mL of ethyl acetate (EtOAc) was added to each flask to stop the fermentation. The flasks were allowed to stand for 12 h, and the EtOAc solution was evaporated to dryness. The EtOAC extract was reconstituted with 5% aqueous methanol and was extracted with n-heptane to afford a dark orange syrup after evaporation.

### 3.4. Isolation of 1–4

The aqueous methanolic extract (6.1 g) was subjected to silica gel column chromatography and elution was carried out using the following solvent systems: petroleum ether, petroleum ether-EtOAc (4:1, 3:2, 1:1, 2:3, 1:4), EtOAc, dichloromethane (DCM), and DCM-MeOH (4:1, 3:2, 1:1) to yield 10 pooled fractions. Fraction 4 (180 mg) was purified using preparative reversed-phase HPLC using the following gradients: 35% acetonitrile with 0.1% formic acid in water (containing 0.1% formic acid) (0–3 min), followed by a linear gradient to 100% acetonitrile with 0.1% formic acid for 20 min, and an additional 10 min using the latter solvent to afford **1** (t_R_ = 10.0 min) (26.8 mg), **2** (t_R_ = 9.0 min), and **3** (1.65 mg, at t_R_ = 8.0 min). Fraction 6 (390 mg) was also subjected to reversed-phase HPLC using the same gradient conditions to yield **4** (3.4 mg, t_R_ = 5 min).

*(-)–scleroderolide (**1**)*. [a]_D_ −7.5°. ^1^H NMR (500 MHz, Acetone-*d_6_*) δ 14.15 (s, 1H), 6.95 (s, 1H), 4.88 (q, *J* = 6.6 Hz, 1H), 2.72 (s, 3H), 1.57 (s, 3H), 1.54 (d, *J* = 6.6 Hz, 3H), 1.34 (s, 3H). ^13^C NMR (126 MHz, Acetone-*d_6_*) δ 169.9, 169.6, 167.4, 155.9, 144.8, 137.1, 129.8, 122.1, 119.2, 117.3, 108.9, 107.2, 92.8, 43.1, 25.5, 22.3, 20.6, 14.6 [10,11,12]. ESI-MS [M + H]^+^ *m/z* 329.05.

*(-)–sclerodione (**2**)*. [a]_D_ −55.7°. ^1^H NMR (500 MHz, Acetone-*d_6_*) δ 6.72 (s, 1H), 4.72 (q, *J* = 6.6 Hz, 1H), 2.70 (s, 3H), 1.54 (s, 3H), 1.49 (d, *J* = 6.6 Hz, 3H), 1.29 (s, 3H). ^13^C NMR (126 MHz, Acetone-*d_6_*) 187.7, 186.3, 164.3, 155.1, 154.9, 152.8, 145.6, 120.2, 118.9, 109.5, 108.4, 107.1, 92.3, 44.2, 26.0, 22.0, 21.3, 14.7 [11,13,14,15]. ESI-MS [M - H]^−^ *m/z* 310.99.

*(-)–trypethelone (**3**)*. [a]_d_−10.7°. ^1^H NMR (700 MHz, Acetone-*d_6_*) δ 7.35 (d, *J* = 2.5 Hz, 1H), 6.96 (d, *J* = 2.5 Hz, 1H), 4.70 (q, *J* = 6.6 Hz, 1H), 2.57 (s, 3H), 1.47 (d, *J* = 6.7 Hz, 3H), 1.40 (s, 3H), 1.22 (s, 3H). ^13^C NMR (176 MHz, Acetone-*d_6_*) δ 182.7, 175.9, 171.7, 160.9, 141.5, 135.4, 124.5, 122.3, 118.5, 115.9, 92.9, 43.7, 26.1, 22.1, 20.6, 14.9 [14,16]. ESI-MS [M + H]^+^ *m/z* 273.08.

*8-O-4′-Diferulic acid (**4**)*. ^1^H NMR (500 MHz, Acetone-*d*_6_) δ 7.62 (s, 1H), 7.58 (s, 1H), 7.53 (d, *J* = 2.0 Hz, 1H), 7.45 (d, *J* = 2.0 Hz, 1H), 7.43 (s, 1H), 7.24 (dd, *J* = 8.4, 2.0 Hz, 1H), 6.84 (d, *J* = 3.9 Hz, 1H), 6.82 (d, *J* = 3.9 Hz, 1H), 6.44 (d, *J* = 15.9 Hz, 1H), 4.01 (s, 3H), 3.74 (s, 3H)) [17,18]. ESI-MS [M − H]^−^ *m/z* 385.07.

### 3.5. Biological Activity Assays

*α-Glucosidase assay* [24]. The substrates p-nitrophenyl α-glucopyranoside (p-NPG) and α-glucosidase were purchased from Sigma (St. Louis, USA). N-deoxynojirimycin was used as a positive control. The standard assay protocol established by the Enzyme Inhibition Laboratory was adopted with minor modifications. First, 0.85 units/mL enzyme and 0.7mM of substrate were used for the initial screening of the fungal extracts, and 100mM sodium phosphate buffer containing 50 mM NaCl was prepared in deionized water (pH 6.8). The change in the absorbance was monitored initially before incubation and 30 min after the addition of *p*-NPG substrate. Percentage inhibition of enzyme by various test compounds was calculated using the formula:

$$\%\;\mathrm{inhibition}=\;\frac{\left(\mathrm{ABS}\;\mathrm{of}\;\mathrm{Blank}\;-\mathrm{ABS}\;\mathrm{of}\;\mathrm{Sample}\right)}{\mathrm{ABS}\;\mathrm{of}\;\mathrm{Blank}}\times \;100$$

*Pancreatic Lipase Assay* [25]. The lipase inhibitory activity of compounds **1**–**3** was measured using a previously published protocol with minor modifications. Thirty five microliters of each test compound in DMSO, 15 µL of pancreatic lipase enzyme (1 mg/mL buffer), and 45 µL Tris-HCl buffer (2.5 mmol Tris, pH = 7.4 and 2.5 mmol NaCl) were mixed in a 96 well microtiter plate and incubated at 25 °C for 15 min. The enzymatic hydrolysis was started upon the addition of 15 µL p-nitrophenyl butyrate (p-NPB). The number of p-nitrophenolate ions generated by the reaction was monitored using microplate reader, measured before incubation and 30 min after the addition of the substrate with corresponding change in absorbance at 405 nm. IC_50_ values of compounds **1–3** were calculated using the least-squares regression lines of the plots of the logarithm of the sample concentration (log) versus the pancreatic lipase activity (%). The known lipase inhibitor orlistat was used as a positive control (IC_50_ = 0.5 µM).

$$\%\;\mathrm{lipase}=\;\frac{\left(\mathrm{ABS}\;\mathrm{compound}\;\mathrm{with}\;\mathrm{substrate}-\mathrm{ABS}\;\mathrm{compound}\;\mathrm{with}\;\mathrm{substrate}\right)}{\mathrm{ABS}\;\mathrm{of}\;\mathrm{Blank}-\;\mathrm{ABS}\;\mathrm{of}\;\mathrm{Negative}\;\mathrm{Control}}\times \;100$$

The cytotoxic effects of the compounds were evaluated using a standard procedure [23].

### 3.6. Computational Calculations

*Molecular docking studies* [26,27]. Compounds **1** and **2** were subjected to molecular docking simulations with the enzymes pancreatic lipase (PDB ID: 1ETH) and α-glucosidase (PDB ID: 5ZCC) to assess their binding characteristics. The enzymes were fetched from the protein data bank as co-crystallized structures. USCF Chimera (version 1.13.1) was used to facilitate the removal of bound residues and minimization of structures. Dock-prepping of ligand and protein structures was done using Antechamber and molecular docking was performed using the BFGS algorithm of AutoDock Vina (version 1.1.2). Validation of the docking protocol was done via redocking experiment of the co-crystallized ligands. The conformational protein–ligand structure was visualized and analyzed using Biovia Discovery Studios (version 4.1).

*Drug-likeness, ADME, and toxicity prediction* [20]. The computational prediction of the pharmacokinetic (ADME) properties of isolated compounds was done using SwissADME program (Molecular Modeling Group, Swiss Institute of Bioinformatics ©2019, online version). The ORISIS Property explorer program (Thomas Sander, Idorsia Pharmaceuticals Ltd., 2017) was employed for in silico toxicity prediction.

## 4. Conclusions

This study highlighted the first chemical study in the fungal genus *Pseudolophiostoma*, where four compounds, including three phenalenones and one dimeric phenylpropanoid, were isolated and identified. The nor-phenalenone derivatives scleroderolide (**1**) and sclerodione (**2**) were identified as the biologically active constituents, with inhibitory activities against *α*-glucosidase and lipase. The mechanisms of action of each compound were validated through in silico studies and showed strong affinities to both target enzymes. The in silico ADMETox-calculated properties for both compounds were encouraging, and these compounds should thus be considered for further investigations. Our findings, overall, are significant and establish the potential of phenalenones **1** and **2** as leads for diabetes and obesity drug discovery.

## Figures and Tables

**Table 1:** α-Glucosidase- and lipase-inhibitory activities of compounds **1–4.**

Sample	Vs. α-glucosidase IC_50_ (µM)	Vs. lipase IC_50_ (µM)
**1**	48.7	3.4
**2**	120.0	1.0
**3**	>100	>100
**4**	nt	nt
N-deoxynojirimycin	130.5	-
Orlistat	-	9.4

nt = not tested.

**Table 2:** Lipinski’s Rule of Five for absorption, distribution, metabolism, and excretion (ADME) analysis of isolated compounds.

**Compound**	**Lipinski’s Rule of Five**					**Drug-Likeness**
	**Molecular Weight (g/mol)**	**Lipophilicity (MLogP)**	**H-bond Donors**	**H-bond Acceptors**	**Rule Violations**	
	**<500**	**<5**	**<5**	**<10**	**<2**	
**1**	328.32	1.40	2	6	0	yes
**2**	312.32	1.13	2	5	0	yes
**3**	272.30	1.06	1	4	0	yes

**Table 3:** Predicted toxicity parameters of compounds **1**–**3**.

	1	2	3
Mutagenicity	High risk	Medium risk	None
Tumorigenicity	High risk	High risk	None
Irritant Effect	None	None	None
Reproductive Toxicity	None	None	High risk
cLogP	2.62	2.88	2.44
Water Solubility	−4.91 (moderately soluble)	−5.29 (moderately soluble)	−3.34 (soluble)

**Table 4:** Cytotoxicity of compounds **1**–**3**.

Compound	IC_50_ vs. L929 (μg/mL)	IC_50_ vs. KB3.1 (μg/mL)
**1**	15	21
**2**	27	18
**3**	2.7	4.2
Epothilone B	9 × 10^−4^	4 × 10^−5^

## Supplementary Materials

The following are available online at https://www.mdpi.com/1420-3049/25/4/965/s1, Figure S1: LC-DAD-ESIMS profile of (-)-scleroderolide (**1**); Figure S2: LC-DAD-ESIMS profile of (-)-sclerodione (**2**); Figure S3: LC-DAD-ESIMS profile of (-)-trypethelone (**3**); Figure S4: LC-DAD-ESIMS profile of 8-O-4′-diferulic acid (**4**); Table S1: Summary of ligand interactions of compounds **1** and **2** against α-glucosidase and pancreatic porcine lipase.
