# Supplementary material for: Alpha-Glucosidase- and Lipase-Inhibitory Phenalenones from a New Species of Pseudolophiostoma Originating from Thailand
Source: Molecules. 2020 Feb 20;25(4):965. doi: 10.3390/molecules25040965 (PMC7070682; doi:10.3390/molecules25040965)
Supplement: Supplementary file 1 [file molecules-25-00965-s001.pdf]

SUPPLEMENTARY MATERIAL FOR

Alpha-Glucosidase- and Lipase-Inhibitory Phenalenones from a New Species of *Pseudolophiostoma* Originating from Thailand

Allan Patrick G. Macabeo [1,2], Luis Agustin E. Pilapil [1], Katherine Yasmin M. Garcia[1], Mark Tristan J. Quimque [1], Chayanard Phukhamsakda [3], Allaine Jean C. Cruz [1], Kevin D. Hyde [3] and Marc Stadler [2,*]

[1] Laboratory for Organic Reactivity, Discovery and Synthesis (LORDS), Research Center for the Natural and Applied Sciences, University of Santo Tomas, Espana Blvd., 1015 Manila, Philippines

[2] Center of Excellence in Fungal Research, Mae Fah Luang University, Chiang Rai 57100, Thailand

[3] Department of Microbial Drugs, Helmholtz Centre for Infection Research and German Centre for Infection Research (DZIF), partner site Hannover/Braunschweig, Inhoffenstrasse 7, 38124, Braunschweig, Germany

* Correspondence: Marc.Stadler@helmholtz-hzi.de

LIST OF SUPPORTING INFORMATION

	Page
**Figure S1.** LC-DAD-ESIMS profile of (-)-scleroderolide (**1**)	3
**Figure S2.** LC-DAD-ESIMS profile of (-)-sclerodione (**2**)	4
**Figure S3.** LC-DAD-ESIMS profile of (-)-trypethelone (**3**)	5
**Figure S4.** LC-DAD-ESIMS profile of 8-*O*-4'-diferulic acid (**4**)	6
**Table S1.** Summary of ligand interactions of compounds **1** and **2** against *α*-glucosidase and pancreatic porcine lipase	7

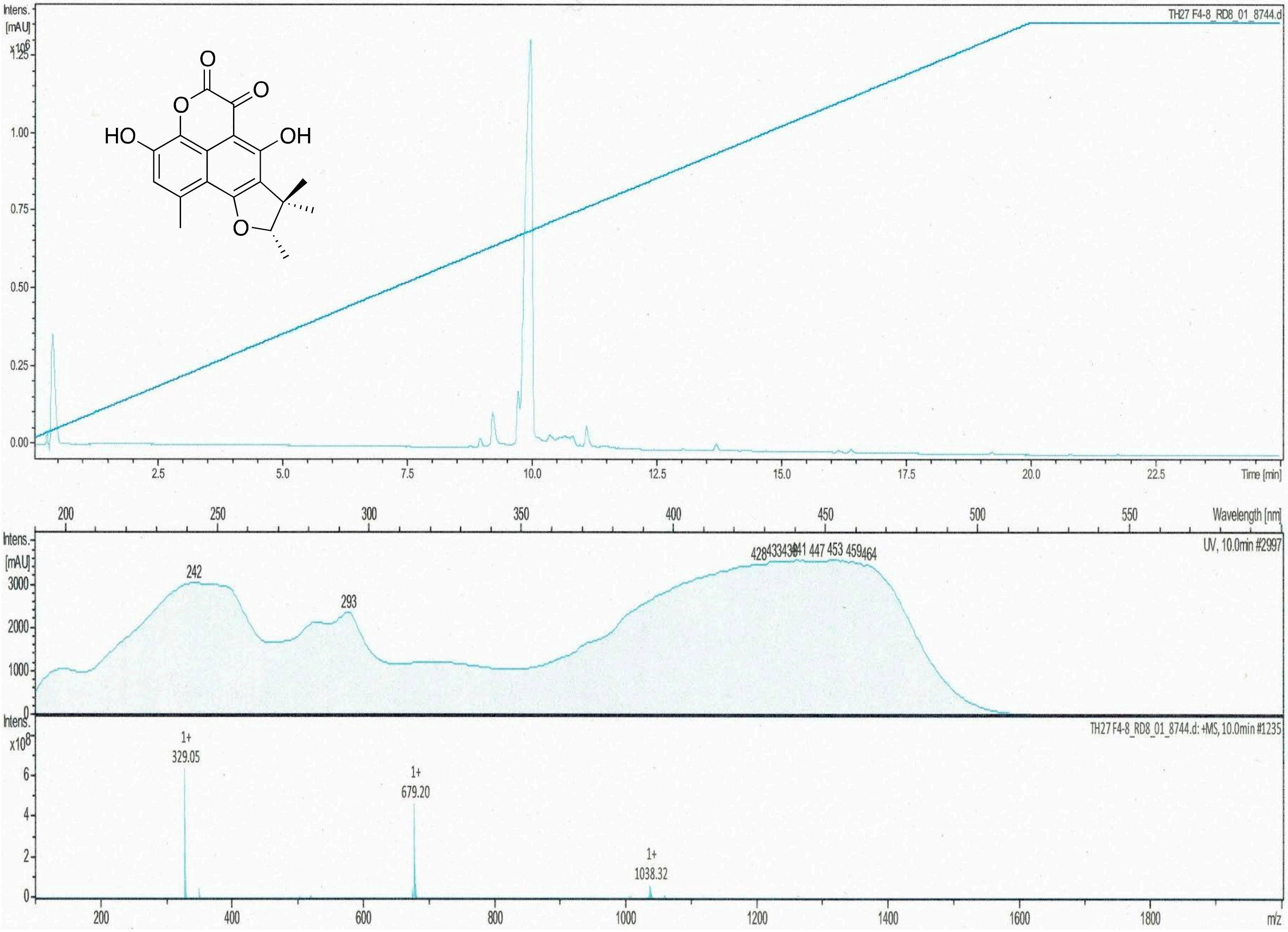

Intens.
[mAU]
x10^6
TH27 F4-8_RD8_01_8744.d
HO
OH
O
Time [min]
Wavelength [nm]
UV, 10.0min #2997
242
293
428 433 436 441 447 453 459 464
TH27 F4-8_RD8_01_8744.d: +MS, 10.0min #1235
x10^8
1+
329.05
1+
679.20
1+
1038.32
m/z

Figure S1. LC-DAD-ESIMS profile of (-)-scleroderolide (**1**).

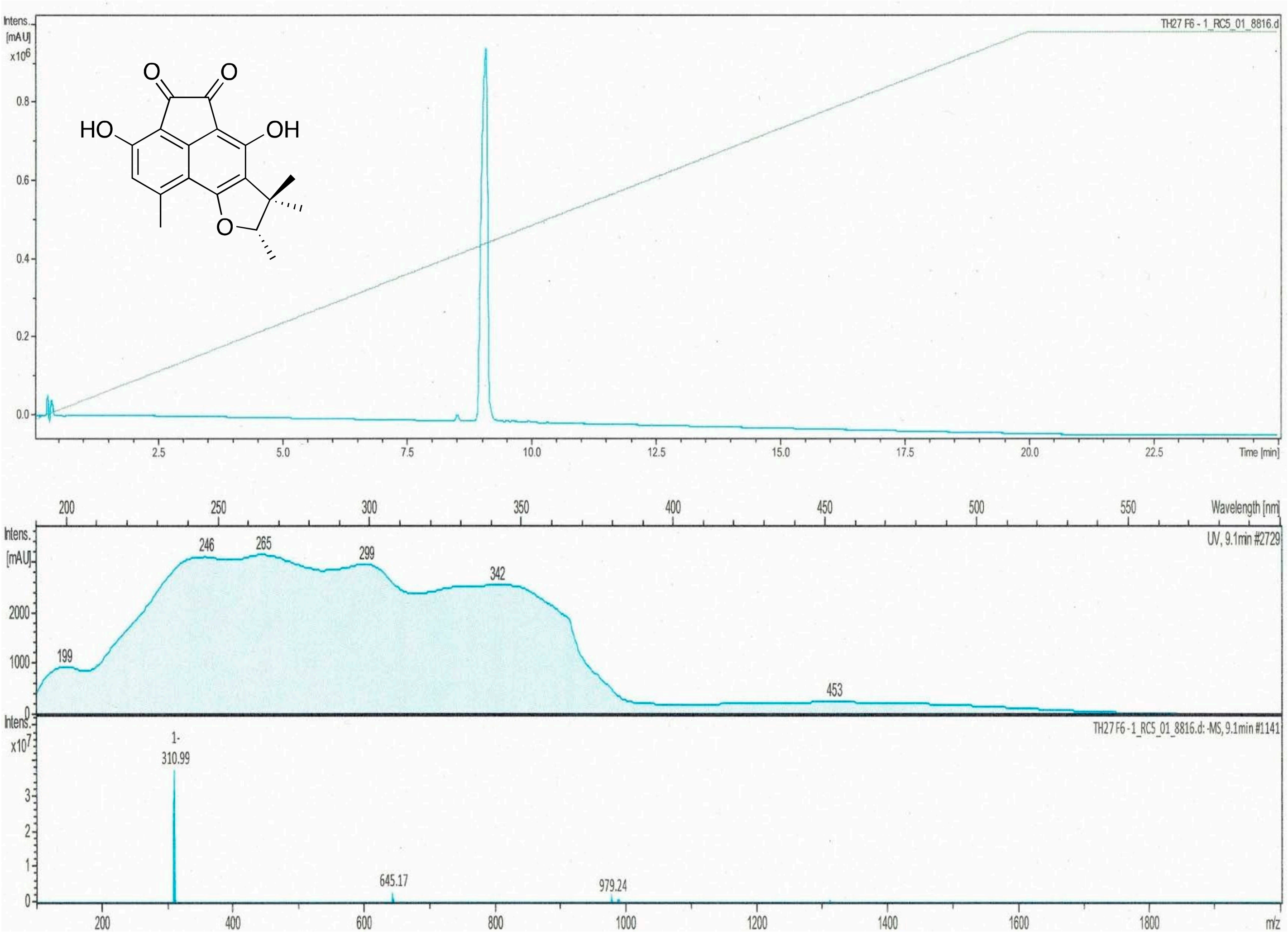

Figure S2. LC-DAD-ESIMS profile of (-)-sclerodione (**2**).

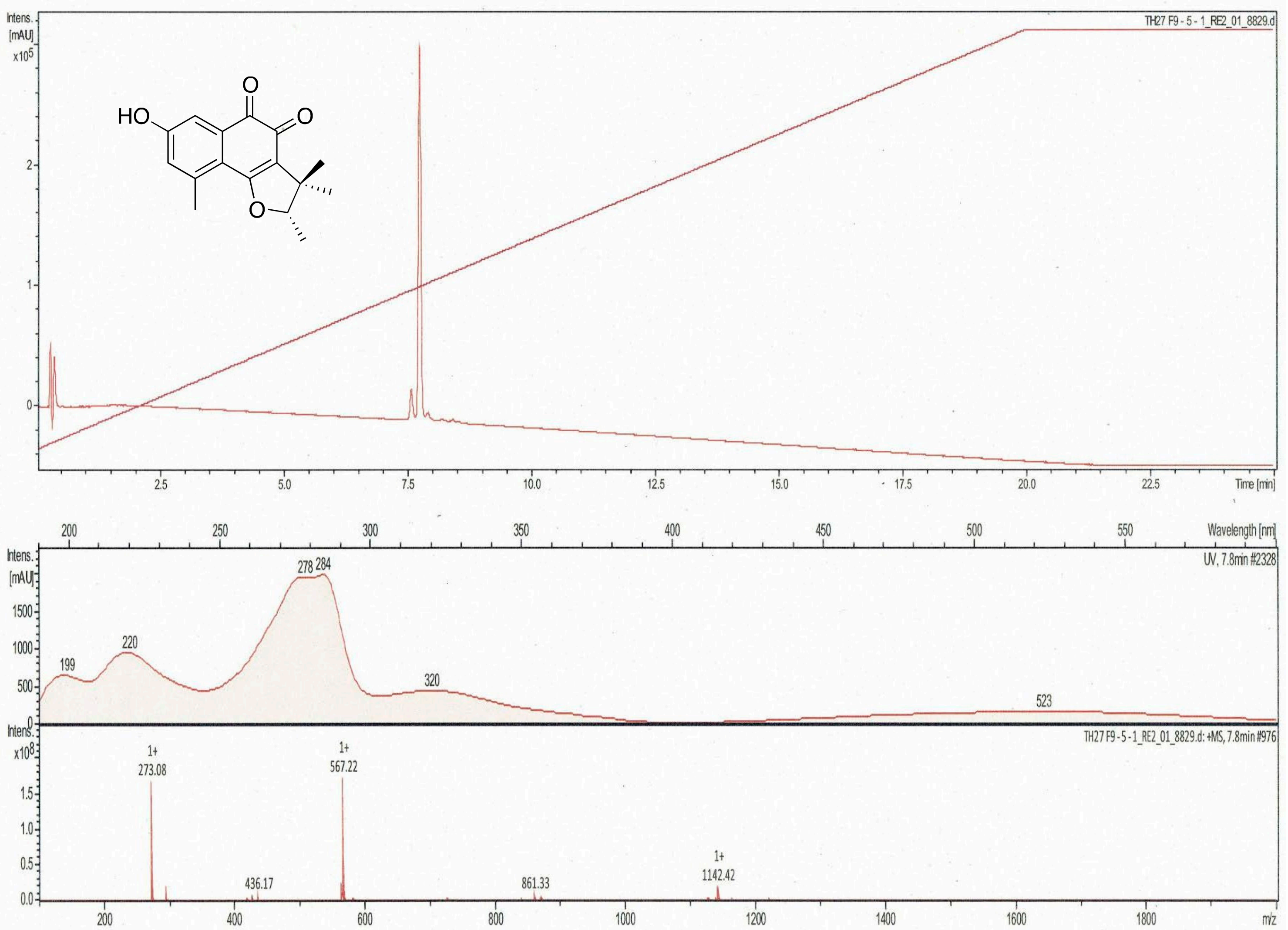
Intens.
[mAU]
x10^5
TH27 F9 - 5 - 1_RE2_01_8829.d
HO
O
O
Time [min]
Wavelength [nm]
UV, 7.8min #2328
199
220
278 284
320
523
TH27 F9 - 5 - 1_RE2_01_8829.d: +MS, 7.8min #976
x10^8
1+
273.08
1+
567.22
436.17
861.33
1+
1142.42
m/z

Figure S3. LC-DAD-ESIMS profile of (-)-trypethelone (**3**).

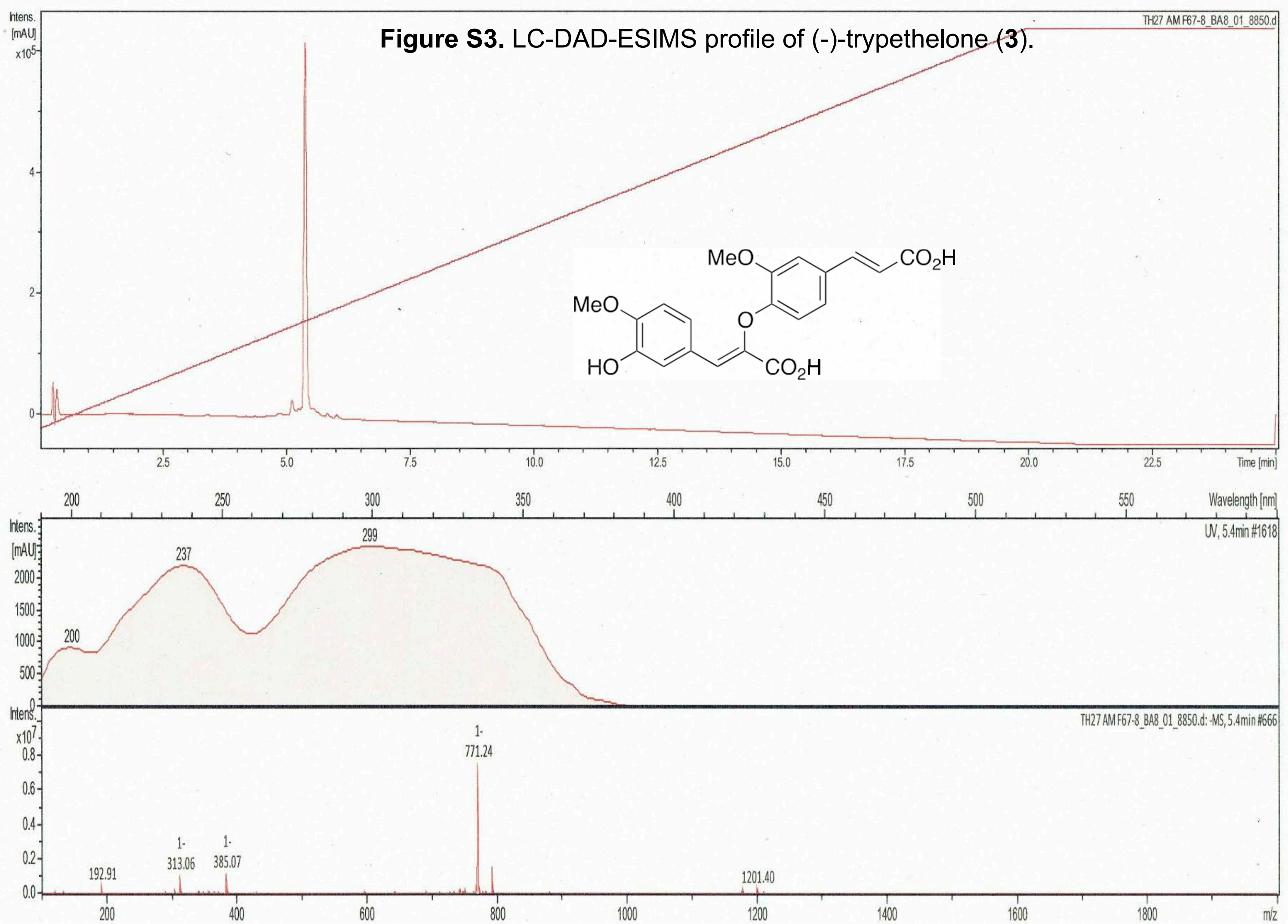

Figure S4. LC-DAD-ESIMS profile of 8-*O*-4'-diferulic acid (**4**).

Table S1. Summary of ligand interactions of compounds **1** and **2** against *α*-glucosidase and pancreatic porcine lipase.

Interaction	Interacting amino acids of pancreatic lipase (1ETH)			Interacting amino acids of α-glucosidase (5ZCCC)		
	**1**	**2**	Orlistat	**1**	**2**	*N*-Deoxy nojirimycin
Conventional hydrogen bond	Ser^{153} Phe^{216}	Ser^{153} His^{264}	Gly^{77} His^{152} Ser^{153}	Gln^{256} Asp^{327} Arg^{411}	Asp^{327} Arg^{411}	Asp^{60} Gln^{167} Gln^{256} Arg^{411}
π Stacking	Phe^{78} His^{264}	Phe^{78} Phe^{216}		Phe^{163}	Phe^{163}	
π-Ion				Asp^{327}	Asp^{327}	
Salt bridge						Phe^{163} Asp^{199} Asp^{327}
π-Alkyl/alkyl	Ala^{179} Pro^{181}	Ile^{210} Val^{260} Ala^{261}	Ile^{79} Ile^{210} Pro^{181} Trp^{253} Arg^{257}	Ile^{143} Phe^{144} Phe^{225}	Ile^{143} Phe^{144} Phe^{225}	
π-Donor hydrogen bond		Tyr^{115}	Tyr^{115}			
π-σ	Tyr^{115}					
